# Quality of life of patients with allergic rhinitis at a South African referral hospital: a prospective cross-sectional study

**DOI:** 10.11604/pamj.2021.40.193.28714

**Published:** 2021-12-01

**Authors:** Devesh Ramdhani, Tamaryn Daniller, Riaz Yakoob Seedat

**Affiliations:** 1 Department of Otorhinolaryngology, Universitas Academic Hospital and University of the Free State, Bloemfontein, South Africa

**Keywords:** Quality of life, allergic rhinitis, nasal sprays

## Abstract

Allergic rhinitis causes nasal as well as extranasal symptoms, and may adversely affect quality of life. The aims of this study were to determine the impact of allergic rhinitis on the health-related quality of life of adult patients attending the Ear Nose and Throat clinic at Universitas Academic Hospital, a public referral hospital, in Bloemfontein, South Africa, and to determine the change in the health-related quality of life of patients with allergic rhinitis after one month of treatment. This was a prospective cross-sectional study of adult patients who were newly diagnosed with allergic rhinitis. Patients completed the Mini Rhinoconjunctivitis Quality of Life Questionnaire (MiniRQLQ) at initial presentation and at follow-up after one month of appropriate treatment. Eighty-five (85) patients were included. At the initial visit, patients were most troubled by activity limitations and nasal symptoms, with a blocked nose being the most troublesome nasal symptom. There was a statistically significant improvement in scores of all domains after one month of treatment, with the mean total symptom score improving from 2.05±0.73 to 0.94±0.49. Allergic rhinitis had a negative impact on quality of life of patients. Appropriate treatment resulted in an improvement in quality of life.

## Introduction

Allergic rhinitis is a condition due to an IgE-mediated allergic reaction that results in nasal (nasal obstruction, rhinorrhoea, post-nasal drip, sneezing, and nasal itching) as well as extranasal symptoms [1]. These include itching of the eyes and palate, conjunctival irritation and erythema, snoring and sleep disturbance. Lack of adequate sleep may lead to irritability, fatigue, memory deficits, daytime somnolence, and impaired cognitive performance [2]. Patients with allergic rhinitis may experience difficulties with learning, impaired work productivity and may be excluded from social activities. They also experience practical problems such as having to carry around tissues. The socioeconomic status of patients can be impacted due to the cost of treatment and poor work productivity. All these factors can affect the quality of life (QoL) of patients with allergic rhinitis.

QoL can be defined as the subjective value a person places upon satisfaction with his or her life [3]. Health-related quality of life (HRQoL) has been defined as the functional effects of an illness and its consequent therapy upon a patient, as perceived by the patient [3]. Instruments used to measure HRQoL include generic instruments such as the Medical Outcomes Study 36-Item Short-Form Health Survey (SF-36) and Kidscreen-27, as well as disease-specific instruments, such as the Rhinoconjunctivitis Quality of Life Questionnaire (RQLQ) and Nasal Comfort Index [3]. The RQLQ is a validated questionnaire that consists of a 28 item list in health-related domains (practical problems, nasal symptoms, ocular symptoms, sleep activities, and emotional function) [4].

The MiniRQLQ is an abbreviated version of the RQLQ that has 14 items across five domains (activities, practical problems, nose, eye and other symptoms) [5]. Each item is scored for the preceding week on a scale from 0 (not troubled) to 6 (extremely troubled). The overall and domain specific scores are the mean of all scores for all domain scores. The questionnaire has been validated for use in South Africa in English and Afrikaans. Most studies on QoL of patients with allergic rhinitis have been carried out in developed countries, with only a few studies having been carried out in African countries [6-8]. The objectives of this study were to determine the impact of allergic rhinitis on the QoL of adult patients attending the Ear Nose and Throat clinic at Universitas Academic Hospital, a public referral hospital, in Bloemfontein, South Africa, using the MiniRQLQ and to determine the change in the QoL of patients with allergic rhinitis after one month of treatment.

## Methods

This was a prospective cross-sectional study of adult patients (18 years and older) who were newly diagnosed with allergic rhinitis based on their symptoms, examination findings and positive skin prick tests for common aeroallergens at the Department of Otorhinolaryngology at Universitas Academic Hospital over a one-year period, between 1 May 2017 and 30 April 2018. All patients who met the inclusion criteria during the time period of the study were included. Demographic data and existing treatment were recorded at the initial visit. Patients completed the MiniRQLQ at initial presentation and at the follow-up visit after one month of appropriate treatment. We attempted to reduce bias by using a standardized, validated questionnaire. The number of patients diagnosed with allergic rhinitis during the study period determined the sample size.

Written informed consent was obtained from all patients. The study was submitted and approved by the Health Sciences Research Ethics Committee of the University of the Free State (HSREC 51/2017) and Free State Department of Health (FS_2017RP52_91). Descriptive statistics, namely frequencies and percentages for categorical data and means and standard deviations for numerical data, were calculated. Scores were compared using the t-test. A p-value less than 0.05 was considered statistically significant.

## Results

There were 85 patients newly diagnosed with allergic rhinitis in the time period. All these patients were included in the study. There were 30 (35.3%) males and 55 (64.7%) females. The mean age of patients was 37.9 years (range 18-77 years). At the time of their first visit to the ENT clinic, 36 (42.4%) of the participants were on treatment for nasal symptoms. All 36 (100%) of these patients were on intranasal steroids. Eight (22.2%) were also on an oral antihistamine, and nine (25%) were also using a topical decongestant. During this visit, patients were most troubled by activity limitations and nasal symptoms (Table 1), with a blocked nose being the most troublesome nasal symptom (Table 2).

**Table 1:** mean scores pre- and post-treatment

Question group	Pre-treatment mean score ± SD	Post-treatment mean score ± SD	Change in mean score (95% CI)	P-value (Cohen’s d)
Activity limitations	3.16±1.05	1.77±0.84	-1.63,-1.15	<0.001 (1.25)
Practical problems	2.13±1.34	1.00±0.78	-1.40,-0.86	<0.001 (0.91)
Nasal symptoms	2.64±1.10	1.31±0.82	-1.55,-1.11	<0.001 (1.30)
Eye symptoms	1.46±0.94	0.36±0.54	-1.29,-0.92	<0.001 (1.30)
Other symptoms	0.87±0.98	0.23±0.64	-0.77,-0.49	<0.001 (0.92)
Overall	2.05±0.73	0.94±0.49	-1.26,-0.96	<0.001 (1.61)

**Table 2:** scores for all questions at initial and follow-up visits

Question	Pre-treatment mean score±SD	Post-treatment mean score±SD	Change in mean score (95% CI)	P-value (Cohen's d)
Regular activities at home and at work	3.26±1.03	1.78±0.98	-1.73,-1.24	<0.001 (1.29)
Recreational activities	2.92±1.16	1.82±0.99	-1.37,-0.81	<0.001 (0.85)
Sleep	3.32±1.42	1.73±0.98	-1.91,-1.27	<0.001 (1.08)
Need to rub nose/eyes	2.31±1.44	1.14±0.86	-1.47,-0.86	<0.001 (0.82)
Need to blow nose repeatedly	1.95±1.64	0.86±0.86	-1.42,-0.77	<0.001 (0.72)
Sneezing	2.21±1.51	1.39±1.12	-1.08,-0.57	<0.001 (0.70)
Blocked nose	3.91±1.25	1.82±1.14	-2.40,-1.77	<0.001 (1.43)
Runny nose	1.80±1.70	0.73±0.91	-1.42,-0.72	<0.001 (0.65)
Itchy eyes	1.88±1.39	0.45±0.75	-1.76,-1.12	<0.001 (0.96)
Sore eyes	0.33±0.92	0.18±0.47	-0.31,0.01	0.06 (0.20)
Watery eyes	2.18±1.47	0.46±0.85	-1.72,1.19	<0.001 (1.14)
Tiredness and/or fatigue	1.86±1.39	0.40±0.93	-0.45,-0.07	<0.001 (1.19)
Thirst	0.46±1.18	0.20±0.74	-0.30,-0.01	0.01 (0.30)
Feeling irritable	0.28±0.97	0.13±0.55	-1.63,-1.15	0.04 (0.23)

All patients were treated with an intranasal corticosteroid (fluticasone) spray. In addition, 79 (92.9%) patients received an oral antihistamine (cetirizine). All patients attended the follow-up visit at a median of 33 days (IQR 13 days). There was a statistically significant improvement in scores of all domains at the follow-up visit (Table 1). The scores for all questions except for sore eyes (Table 2) improved significantly at the follow-up visit.

There were no significant differences in the total symptom score at the initial visit between those who were not on treatment and those who were on treatment (2.0 vs. 2.1, p=0.844). Patients in both the groups, those who were not on treatment at the initial consultation (2.0 vs. 0.9, p<0.001) and those who were on treatment (2.1 vs 1.0, p<0.001) had a significant improvement in the total symptom scores.

## Discussion

AR adversely affected the QoL of patients in our study and an improvement in QoL was observed after one month of treatment. The change in the mean scores was statistically significant with a large effect size for the total score as well as for the scores of all domains. The greatest improvements were in the domains´ activity limitations and nasal symptoms. Patients had a significant improvement in QoL even if they were already on treatment at the time of initial consultation. This highlights the importance of counselling regarding the correct use of treatment.

The most bothersome symptom on initial presentation was a blocked nose, which also showed the greatest improvement. Relief of nasal congestion has the greatest correlation with improvement in HRQoL [6]. As HRQoL scores use arbitrary scales and lack easily interpreted units, a minimal important difference (MID) is necessary to interpret statistically significant results. The MID denotes the smallest significant change in perspective to initiate a change in treatment modality. The minimal clinically significant difference for the MiniRQLQ is 0.7 [9], which is lower than the improvement in the total symptom score.

There are few studies that have studied QoL of patients with allergic rhinitis in Africa. A community based cross-sectional study from Nigeria on HRQoL using the MiniRQLQ showed that allergic rhinitis had a significant effect on the patients´ HRQoL compared to matched controls [7]. The symptom scores in this study were higher than those of our study across all domains. This is unexpected as our study only included patients who were referred for specialist treatment and would therefore be expected to have more severe symptoms.

A Rwandan study on children and adolescents found the most bothersome symptom in children was sneezing, while in adolescents, having to rub their eyes and nose was most troublesome [8]. In contrast, nasal obstruction was the symptom with the highest score in our study.

In a multicentre study conducted in South Africa, allergic rhinitis was found to impair the HRQoL as measured using the RQLQ, with a significant improvement in scores with triamcinolone nasal spray as compared to placebo [6]. Relief of nasal congestion has the greatest correlation with improvement in HRQoL. The most bothersome symptom on initial presentation in our study was a blocked nose, which also showed the greatest improvement with treatment.

A study in South African medical students found that allergic rhinitis affected their quality of life, with class attendance and participation in outdoor activities being affected [10]. Many participants reported that they frequently felt tired or miserable as a result of their nasal symptoms.

A limitation of this study is that it was carried out at a specialist referral clinic. Patients seen at this clinic are therefore more likely to be troubled by their symptoms. The findings may therefore not be generalizable to all patients with allergic rhinitis. However, this study highlights the impact of allergic rhinitis on quality of life and the improvement in quality of life resulting from treatment of allergic rhinitis.

## Conclusion

Allergic rhinitis had a negative impact on quality of life, sleep, daily activities, mental status, and social functioning and treatment together with counselling regarding its use resulted in an improvement in quality of life.

### What is known about this topic

- Allergic rhinitis causes nasal and extranasal symptoms;
- These symptoms may affect quality of life in patients with allergic rhinitis.

### What this study adds

- Allergic rhinitis adversely affected quality of life of adult patients with allergic rhinitis;
- Patients were most troubled by activity limitations and nasal symptoms, with a blocked nose being the most troublesome nasal symptom;
- Appropriate medical treatment improved quality of life in patients with allergic rhinitis with a statistically significant improvement in scores of all domains of the Mini Rhinoconjunctivitis Quality of Life Questionnaire (MiniRQLQ).
